# COVID-19 needs no passport: the interrelationship of the COVID-19 pandemic along the U.S.-Mexico border

**DOI:** 10.1186/s12889-022-13513-1

**Published:** 2022-05-31

**Authors:** John N. Filosa, Adrian Botello-Mares, David Goodman-Meza

**Affiliations:** 1 David Geffen School of Medicine, University of California, Los Angeles (UCLA), 10833 Le Conte Ave, Los Angeles, CA 90095-1688 USA; 2 Department of Population Studies, Colegio de la Frontera Norte, Sonora Nogales, Mexico; 3 Division of Infectious Diseases, David Geffen School of Medicine, University of California, Los Angeles (UCLA), CA Los Angeles, USA

**Keywords:** COVID-19, U.S.-Mexico border, Standardized mortality, Human development index, Vaccination

## Abstract

**Objectives:**

To investigate the impact of the COVID-19 pandemic along the U.S.-Mexico border region and evaluate the relationship of COVID-19 related mortality, socioeconomic status, and vaccination.

**Methods:**

We used indirect standardization to age-adjust mortality rates and calculate standardized mortality ratios [SMR] in both countries. To examine the impact of socioeconomic factors, we calculated the Human Development Index (HDI) by county/municipality. We performed linear regression to understand the relationship between mortality, vaccination, and HDI. We used choropleth maps to visualize the trends seen in the region.

**Results:**

Between January 22nd, 2020 and December 1st, 2021, surges of cases and deaths were similar in dyad cities along the U.S.-Mexico border visualizing the interconnectedness of the region. Mortality was higher in U.S. counties along the border compared to the national average (SMR 1.17, 95% CI 1.15–1.19). In Mexico, border counties had a slightly lower mortality to the national average (SMR 0.94, 95% CI 0.93–0.95). In U.S. border states, SMR was shown to negatively correlate with human development index (HDI), a socioeconomic proxy, resulting in a higher SMR in the border region compared to the rest of the counties. Conversely in Mexican border states, there was no association between SMR and HDI. Related to vaccination, U.S. counties along the border were vaccinated at a greater percentage than non-border counties and vaccination was negatively correlated with HDI. In Mexico, states along the border had a higher ratio of vaccinations per person than non-border states.

**Conclusions:**

The U.S.-Mexico border is a divide of incredible importance not only to immigration but as a region with unique social, economic, environmental, and epidemiological factors that impact disease transmission. We investigated how the COVID-19 pandemic followed trends of previously studied diseases in the corridor such as tuberculosis, HIV, and influenza H1N1. These data state how targeted intervention along the U.S.-Mexico border region is a necessity when confronting COVID-19 and have implications for future control of infectious diseases in the region.

## Background

The U.S.-Mexico border is a divide of incredible importance. Each day, $1.7 billion dollars of goods and services traverses the two countries in addition to hundreds of thousands of people, cars, trucks, and rail containers [1]. Much of the attention the geographic corridor receives revolves around immigration, both legal and illegal. However, the U.S.-Mexico border is a complex region with unique social, economic, and environmental qualities. Residents in this area are often at a disadvantage in terms of education, socioeconomic status, income inequality, and healthcare access that leads to poorer health outcomes [2]. SARS-CoV-2 is an RNA virus in the Coronaviridae family that leads to Coronavirus Disease 2019 (COVID-19) and caused a global pandemic since early 2021 [3]. COVID-19 is transmitted primarily through respiratory droplets and aerosols [4]. The disease’s clinical manifestations can range from an asymptomatic carrier state, a non-specific febrile illness, mild respiratory disease, or acute hypoxic respiratory failure that may lead to death [5]. Little has been reported on the epidemiology of COVID-19 along the U.S.-Mexico border.

The unique factors at the U.S.-Mexico border established the region as an important point of infectious disease dynamics before the COVID-19 pandemic began surfacing throughout the world. For example, assessments of tuberculosis genotype clusters from 2005 to 2010 revealed border areas as regions of high prevalence with spread between U.S.-born and Mexico-born persons [6]. Characteristics of the U.S.-Mexico border have shaped residents’ vulnerability to HIV as well. Regions bordering the U.S. such as Baja California have consistently had a higher HIV/AIDS incidence than the national average [7]. The example most prominent in recent memory, though, is the 2009 H1N1 pandemic. The influenza season of 2009–2010 yielded another iteration of the notorious H1N1 influenza strain likely originating in swine from Mexico. Patients 1 and 2 of the outbreak were both from border counties (San Diego and Imperial respectively) [8]. Though there do not seem to be any population studies examining H1N1 cases throughout the border, there have been retrospective pieces on how the border highlighted existing inequalities [9].

Socioeconomic determinants of health have contributed significantly to many public health emergencies throughout history and are being revisited during the COVID-19 pandemic [10]. There is ongoing research and evidence that the COVID-19 pandemic displays previously known trends of disproportionately impacting marginalized economic and racial groups [11–14]. Similar trends may be occurring during the COVID-19 pandemic, yet, little to no information has been published to highlight the regional epidemic along the U.S.-Mexico border [15]. To investigate this hypothesis, we compared COVID-19 case rates, mortality data, and vaccination data from U.S.-Mexico border and non-border regions, and tested the relationship of COVID-19 mortality with a socioeconomic indicator, the Human Development Index. We then contrasted disparities in these epidemiologic markers, discussed the interrelationship of the COVID-19 epidemic on both sides of the border, and explored potential reasons of differing outcomes along the border.

## Methods

### Data sources

This was an ecological analysis at the county/municipal level for the U.S.-Mexico border region. We extracted data from the COVID-19 Data Repository at Johns Hopkins University [16], U.S. National Center for Health Statistics [17], and the Mexican Ministry of Health’s Department of Epidemiology (*Dirección General de Epidemiología*) [18] for COVID-19 case and death counts. The data we extracted ranged from the beginning of the pandemic to December 1st, 2021. The data included county level U.S. data with confirmed, active, and recovered cases, deaths, the incident rate, and the case fatality ratio (recorded deaths/cases). The Mexican Ministry of Health dataset includes health unit, institution, care location, sex, birth date, residence, municipality, and many others. We extracted sociodemographic indicators from the American Community Survey 2019 (ACS) [19] and the Mexican National Institute of Statistics and Geography (Instituto Nacional de Estadísticia y Geografía) 2020 (INEGI) [20]. We obtained U.S. vaccination data by county from the Centers for Disease Control Data Repository [21], which was updated daily. These data included vaccination numbers and percentages for single and complete vaccination series, and stratification by age groups. The Mexican vaccination data was obtained via scrapping the Mexican Health Secretary’s (Secretaría de Salud) website [22]. All data was downloaded as aggregates at the county/municipal level and was determined to be non-human subjects research by the UCLA Institutional Review Board (IRB).

### Measures

To examine the impact of socioeconomic factors, we calculated the Human Development Index (HDI) by county/municipality. The HDI was created by the United Nations Development Program (UNDP) [23]. HDI is a measure which assesses health, knowledge, and standard of living and compiles them into a singular score. The output of standardized scores then can allow researchers to compare various policies, programs, and events and their relation to socioeconomic factors. ACS provided information on lifespan, high school achievement, and average income. INEGI provided information on lifespan, years of schooling, and average income. We calculated HDI by first creating dimension indices which scale various factors between 0 and 1. The bounds as defined by UNDP are 20–85 years for life expectancy, 0–18 years for expected schooling or 0–15 years for mean schooling, and 100 to 75,000 for GNI per capita. Based on the available data for education systems, we calculated the U.S. and Mexican HDI differently [24]. The U.S. educational index was calculated by weighting for high school achievement. The Mexican educational index was calculated by weighting for average number of school years attained. Given these bounds, the data from ACS and INEGI was scaled between 0 and 1. After scaling, the geometric mean of the 3 outputs was calculated resulting in the final HDI metric.

COVID-19 cases and deaths were based on definitions from the originating data sources. In the U.S., the COVID-19 Data Repository at Johns Hopkins University aggregated data from local health jurisdictions at state and county levels. Local health jurisdictions report cases based on U.S. Centers for Disease Controls and Prevention (CDC) guidelines [25]. As such, positive COVID-19 cases are defined as either a laboratory-confirmed case or a probable case when a case either (1) meets clinical criteria and epidemiologic evidence with no confirmatory lab testing performed for COVID-19, (2) meets presumptive lab evidence and either clinical criteria or epidemiologic evidence, or (3) meets vital records criteria with no confirmatory lab testing performed for COVID-19. In Mexico, positive COVID-19 cases were defined as cases that either had a positive confirmatory test, or were classified as positive by epidemiologic association or by a ruling committee (only for deaths) [26].

To compare COVID-19 mortality rates, we standardized rates to adjust for differences in differing age demographics in a population. We created age-adjusted standardized mortality ratios by indirect standardization. Indirect standardization only requires that we know the age-specific mortality rates for the overall population, total number of deaths (or cases) of the study population, and the age structure of the study population [27]. As the JHU data only provides this level of detail (number of cases/number of deaths), indirect standardization was the only feasible method. Age-specific COVID-19 mortality rates were not available to calculate direct standardized rates. Given this information, we performed indirect standardization by multiplying the population size by the age-specific death rate resulting in the expected number of deaths. We then divided the observed number of deaths by the previously calculated product resulting in the standardized mortality ratio (SMR) [28].

### Analysis

We used the R [29] packages epitools [30] and ggplot2 [31] in order to conduct the analysis and visualization for this project. We plotted time series heatmaps of rates by dyads of bordering county/municipality. We divided the data into phases based on seasons and peaks in COVID-19 cases. These sets included the early pandemic (before 6/1/2020), summer ‘20 (6/1/2020–9/1/2020), fall ‘20 (9/1/2020–12/1/2020), winter ‘20-‘21 (12/1/2020–2/1/2021), spring ‘21 (2/1/2021–6/1/2021), and summer and fall ’21 (6/1/2021–12/1/2021). We mapped calculated SMRs and vaccination rates using choropleth maps. We created descriptive statistics to compare the impact of COVID-19 in border and non-border regions of the U.S. and Mexico. These statistics were able to provide numerical context to the visual differences observed in our mapping. We performed linear regression to measure the association of COVID-19 SMR to HDI and vaccination to HDI of the region. We used a pre-defined alpha of < 0.05 for statistical significance.

## Results

We compiled data from the 25 U.S. counties and 40 Mexican municipalities along the border and temporally and cumulatively analyzed the dataset to understand the dynamics of COVID-19 in the region. Between the start of data collection and December 1st, 2021 there were 1,473,977 cases and 47,906 deaths in the binational border region.

**Fig. 1:**
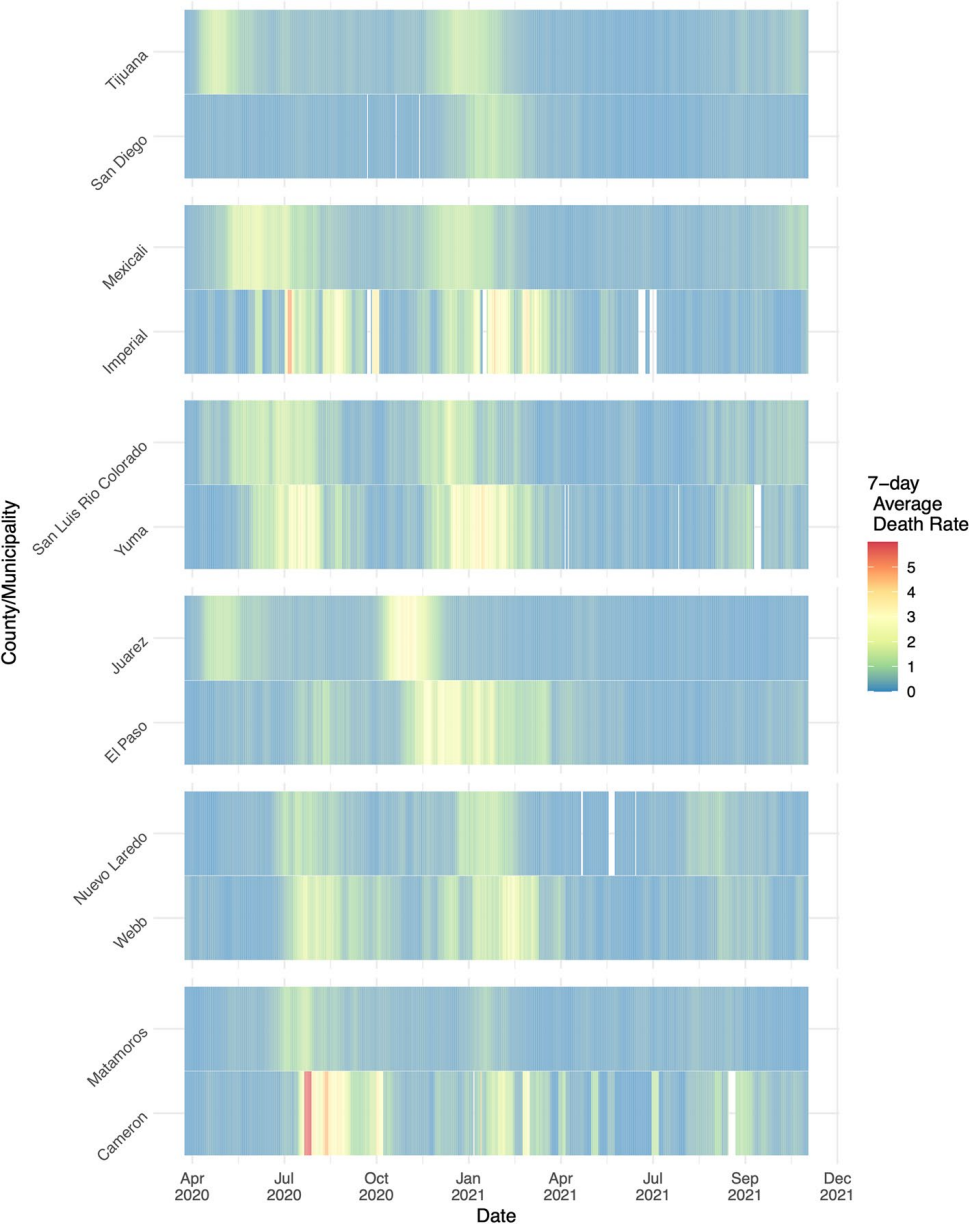
Waves of COVID-19. County/municipality new daily deaths were averaged over a 7-day period, scaled to per 100,000 people, and plotted over time from March 2020 to December 2021

Using weekly death rates scaled to the per capita metric of 100,000 people, we created a heatmap visualizing surges of cases in border counties and municipalities. Neighboring cities experienced lagging peaks of COVID-19 deaths with the preceding peak usually occurring on the Mexican side of the border. This trend occurred both during waves in the spring and summer of 2020 and the fall and winter of 2020–2021 (Fig. 1).

**Fig. 2:**
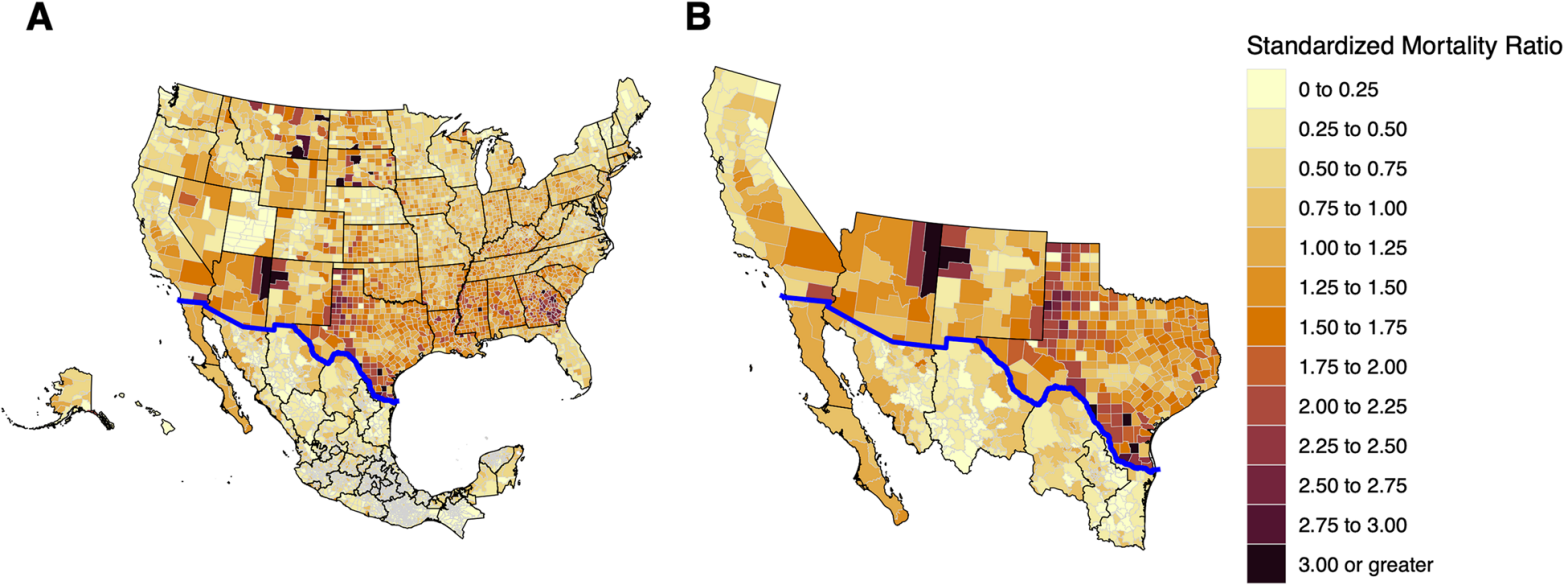
Mapping COVID-19 SMR in the U.S. and Mexico. **A** SMR from COVID-19 was used to create a choropleth map highlighting greater SMR along the border. **B** A zoomed in map of border states

**Fig. 3:**
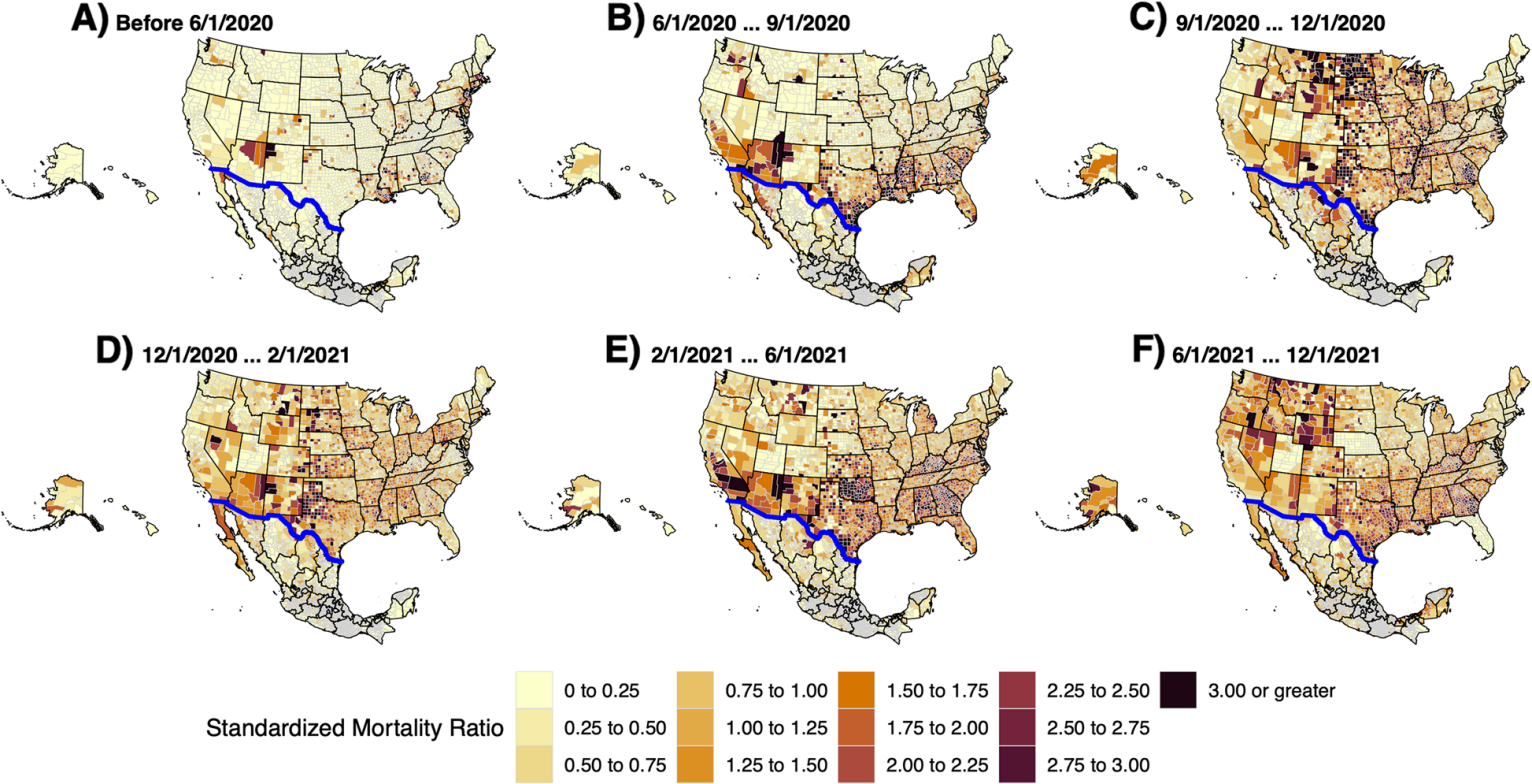
Mapping COVID-19 SMR in the U.S. and Mexico Throughout the Pandemic. SMR from COVID-19 was used to create 6 choropleth maps to highlight how mortality evolved over the course of the pandemic

Using SMR, we created choropleth maps for the U.S. and Mexico by county or municipality. The resulting maps highlighted geographic disparities in the border region compared to non-border regions (Fig. 2). We then created maps by season to visualize the changing hotspots of COVID-19 throughout the pandemic (Fig. 3).

Scaled case rates were similar comparing border to non-border areas in the U.S. (14,857.3 vs. 14,183.6) and Mexico (2,794.5 vs. 3,670.5). However, age-adjusted death rates were greater in border regions both in the U.S. (288.55 vs. 251.37) and Mexico (282.67 vs. 235.17). Additionally, SMR were greater in border regions both in the U.S. (1.17 vs. 1.02) and Mexico (0.94 vs. 0.78) (Table 1). Separating the pandemic into seasons, we found that border regions had a greater SMR compared to non-border regions until approximately February 2021 when the trend reversed (Table 2).

**Table 1:** Comparison of COVID-19 rates by country and border proximity

								95% Confidence interval			95% Confidence interval	
Country	Border Area^a^	Cases	Deaths	Population	Crude Case Rate per 100,000	Crude Death Rate per 100,000	Age-Adjusted Death Rate	Lower	Upper	Standardized Mortality Ratio	Lower	Upper
USA	No	10,096,659	159,416	71,189,008	14,182.90	223.9	251.35	250.12	252.59	1.02	1.01	1.02
	Yes	1,174,581	20,950	7,905,367	14,858.00	265	288.54	284.66	292.48	1.17	1.15	1.18
Mexico	No	860,898	54,807	23,454,811	3,670.50	233.7	235.17	233.21	237.15	0.78	0.78	0.79
	Yes	299,454	26,956	10,715,974	2,794.50	251.5	282.67	279.32	286.06	0.94	0.93	0.95

^a^Only includes counties within states that are on the border

Comparison of COVID-19 SMR by country and border proximity throughout the pandemic

**Table 2:** Comparison of COVID-19 SMR by country and border proximity throughout the pandemic

	Border Area^a^	**Before 6/1/20**	**6/1/20-9/1/20**	**9/1/20-12/1/20**	**12/1/20-2/1/21**	**2/1/21-6/1/21**	**6/1/21-12/1/21**	**All Dates**
USA	No	0.32	1.37	0.82	1	1.85	0.94	1.02
	Yes	0.26	2.48	1.42	1.25	1.85	0.61	1.17
Mexico	No	0.14	0.8	1.13	0.75	0.74	0.91	0.78
	Yes	1.26	1.08	1.49	0.81	0.56	0.63	0.94

^a^Only includes counties within states that are on the border

**Fig. 4:**
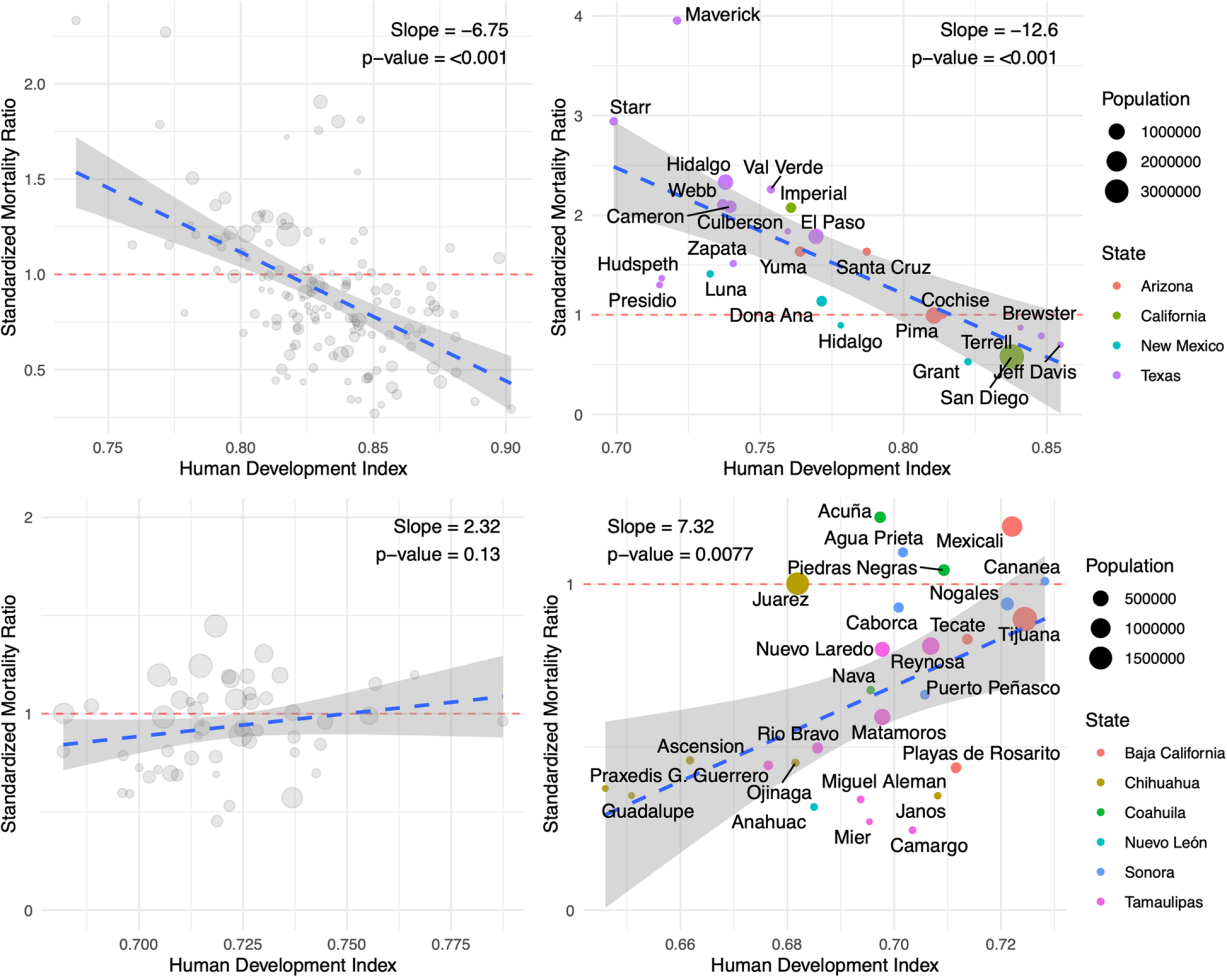
Relationship of COVID-19 SMR and HDI along U.S. and Mexico Border. SMR and HDI were calculated for every county or municipality nationally (left) and in states along the border (right)

Further investigating reasons for the SMR disparities, we plotted HDI against SMR in order to understand correlations between death and socioeconomic conditions. In US border states, there was a negative correlation between SMR and HDI resulting in a higher SMR in the border region compared to the rest of the counties (*p* < 0.001). Conversely in Mexican border states, there was a positive association between SMR and HDI (*p* < 0.01) (Fig. 4).

**Fig. 5:**
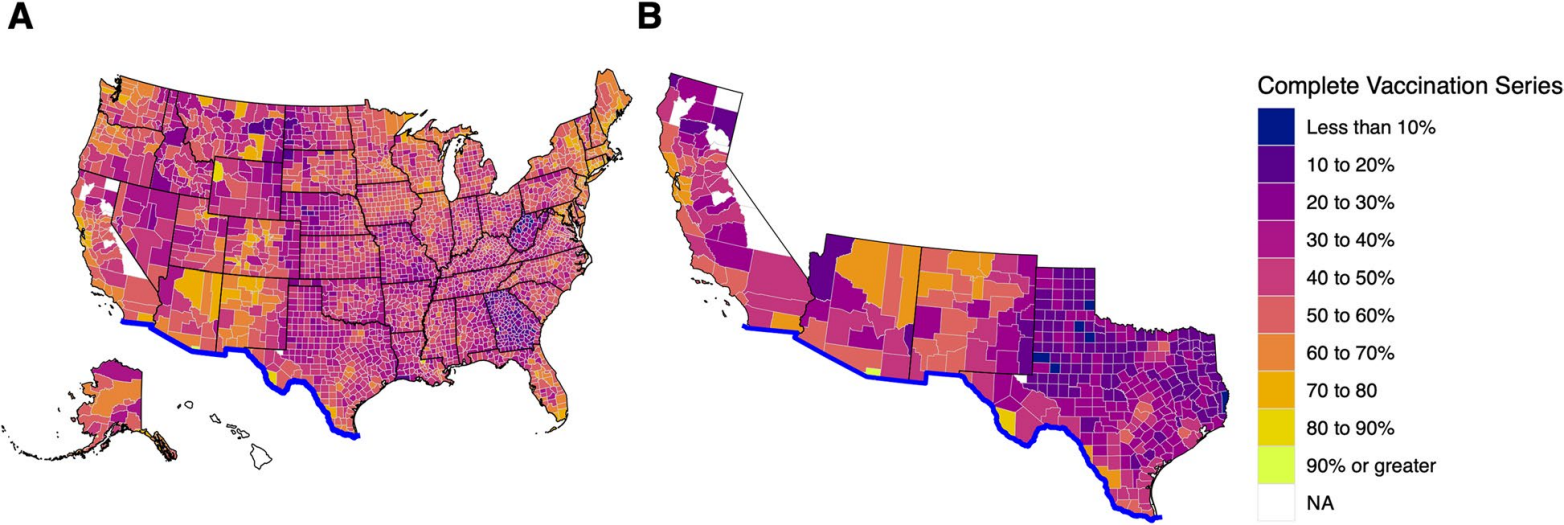
Mapping COVID-19 Vaccination Rates in the U.S. **A** Percent of county residents who have received a complete vaccination series was used to create a choropleth map of the U.S. **B** A zoomed in map of border states. Updated as of 12/1/2021

**Fig. 6:**
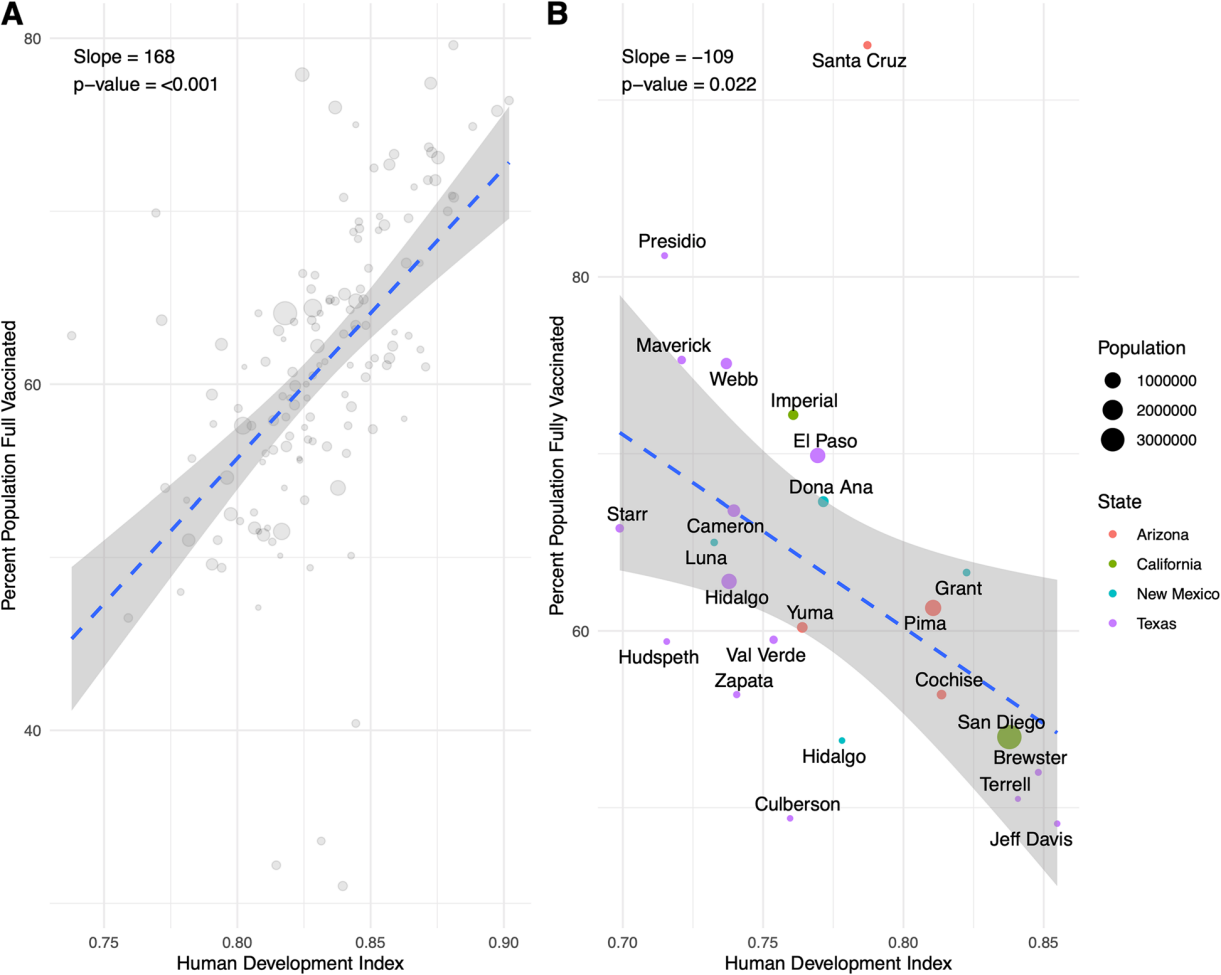
Relationship of COVID-19 Vaccination Rates and HDI in the U.S. Vaccination rates and HDI were calculated for every county nationally (**A**) and in states along the border (**B**)

As the pandemic progressed, vaccination became an important component to combatting the pandemic. We created vaccination maps with choropleth techniques to visualize regions of lower or higher vaccination status (Fig. 5). The resulting maps revealed that U.S. border counties appeared to have higher rates of complete vaccination series compared to non-border counties in those states. Comparing the totals, we found that U.S. border county residents were vaccinated at a higher percentage (61.3% vs. 58.7%) than non-border counties. Comparing counties, those along the border had a higher mean vaccination rate (62.6% vs. 44.4%, *p* < 0.001) than non-border counties. Similar to the regression of HDI against SMR, we plotted the rates for border and non-border counties against HDI which revealed that there was a negative correlation between vaccination rates and HDI (*p* = 0.02) which was opposite to the trend seen nationwide (*p* < 0.001) (Fig. 6).

**Fig. 7:**
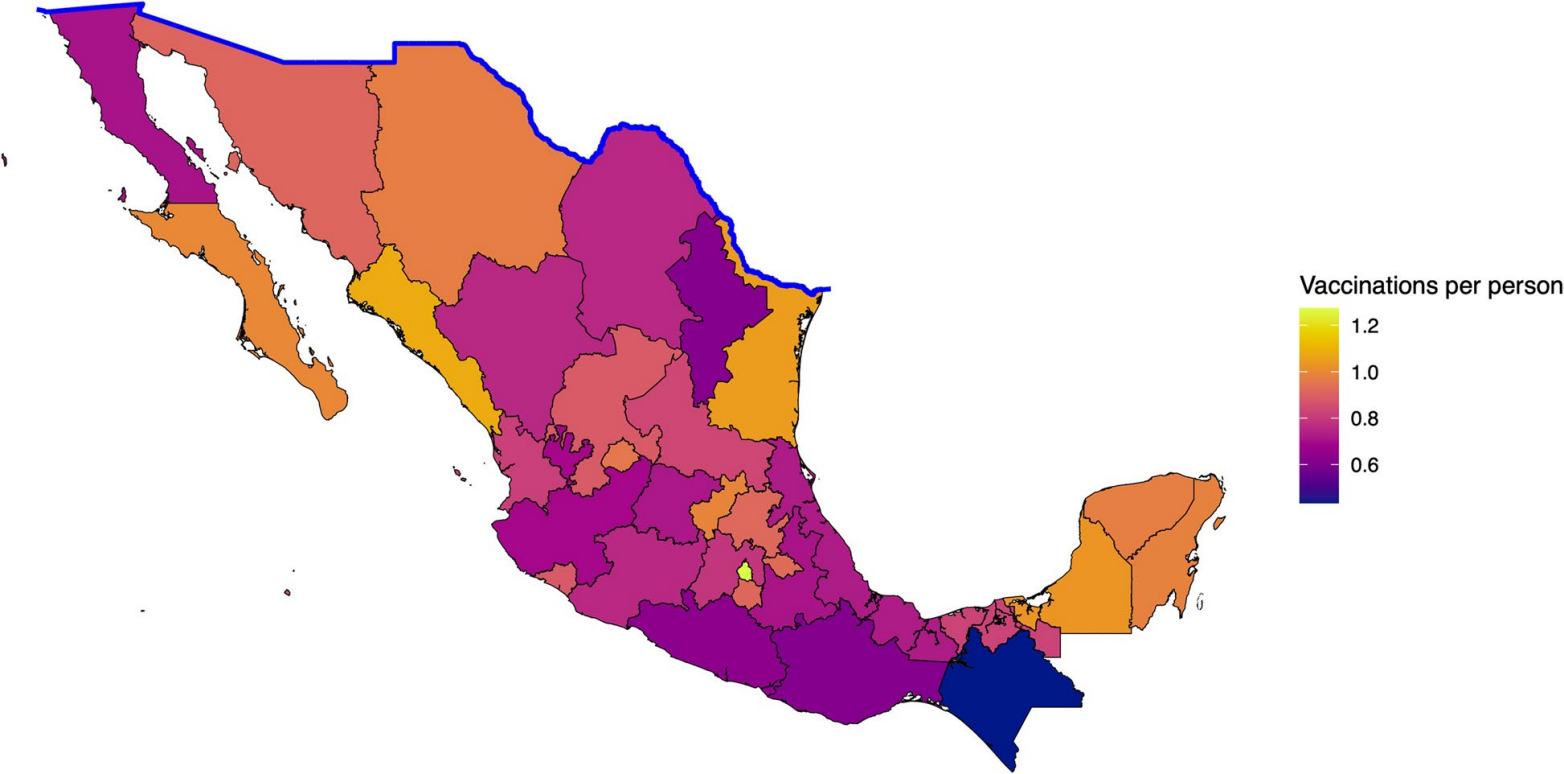
Mapping COVID-19 Vaccination Rates in Mexico. Ratio of vaccinations per person was used to create a choropleth map Mexico. Updated as of 12/1/2021

We also created maps with choropleth techniques to visualize vaccination states of Mexican states (Fig. 7). As data was not disaggregated at the municipality level we did not have the granularity of the U.S. data. Our analysis revealed that in total, border states in Mexico had a higher ratio of vaccinations per person (0.88 vs. 0.81) compared to non-border states. However, there was no significant difference between the mean ratios when comparing border and non-border states (0.88 vs. 0.84, *p* = 0.64).

## Discussion

We analyzed standardized COVID mortality rates along the U.S. Mexico border. We found that along the U.S.-Mexico border region there were higher COVID-19 mortality rates than non-border areas. Despite differing policies on either side of the border, proximal cities experienced linked waves of COVID-19 in terms of peaks and troughs. To inform the disparity between border and non-border areas, we plotted standardized mortality ratio (SMR) against human development index (HDI), a socioeconomic proxy. In US border states, there was a negative correlation between SMR and HDI resulting in a higher SMR in the border region compared to the rest of the counties. However, in Mexican border states, there was no association between SMR and HDI. In the U.S. border states, there was a negative correlation between SMR and vaccination percentage opposite to national trends. Additionally, in both the U.S. and Mexico, border regions were more vaccinated than non-border regions. Our findings overall highlight the importance of targeted regional interventions that consider both sides of the border in unison, and counter the notion that nations can isolate themselves through strict border policies.

Waves of COVID-19 were seen in border cities usually beginning on the Mexican side and surging on the U.S. side shortly after. Reasons for this trend are unclear but may lie in the unidirectional policies that existed surrounding border travel. Starting in March 2020, the U.S. government enacted restrictions on inbound traffic limited to “essential travel”. Reasons deemed as “essential” were citizen or lawful resident return, medical, educational, work, emergency or public health, cross-border trade, government or diplomatic, or military [32]. Conversely, the Mexican government did not impose any inbound travel restrictions [33]. This resulted in U.S. citizens and permanent residents being able to cross the border freely, while Mexican citizens were not able to cross into the U.S. Furthermore, the rates at which state and municipality restrictions were implemented varied between the U.S. and Mexico [34]. In March, California was one of the first states in the U.S. to enact statewide stay-at-home orders, while physical distancing orders in bordering Tijuana and Mexicali were not in effect until weeks later [35]. As initial cases in Mexico were likely imported from the U.S., the heavy interaction between individuals on the Mexican side early in the epidemic lead to Tijuana and Mexicali being early epidemic centers for Mexico like what New York was in the U.S. [35, 36] Later in April, as case counts remained low in California and as restrictions began to lift, cases were likely imported from Mexicali to bordering Imperial County and southern San Diego leading to pressure on hospital centers in those areas. A similar phenomenon occurred on the eastern border leading to high mortality rates in Laredo and Brownsville (Fig. 3).

Reasons for disparities in COVID indicators at the border areas compared to non-border areas are likely sociostructural. The U.S. border region is predominantly populated by Latino individuals and much has been reported on the U.S. side of the increased risk of COVID-19 in Latino and Black communities [37]. Yet, the virus does not have special receptors to target based on race or ethnicity, and it has yet to be proven if there is a particular genetic susceptibility in these racial/ethnic groups that increases these populations’ risk. Hence, the increased risk of disease acquisition is most likely due to sociostructural factors that lead to situations of numerous and prolonged interactions with other individuals that allow for disease transmission. For example, high density housing leads to rapid spread of the virus [38]. Additionally, many individuals due to their documentation status did not qualify for certain economic benefits to mitigate the economic impact of the epidemic. This forced these vulnerable individuals to continue working and be at high-risk for SARS-CoV-2 acquisition. U.S. border counties are amongst the poorest in the country, this tied with high rates of undocumented individuals in these areas, lead to a large segment of the population who lack basic primary and preventative medical care [39]. For mortality outcomes, pre-existing conditions like diabetes or kidney disease play a significant role in Latino communities and has been consistently associated with a higher risk of mortality from COVID [40].

On the Mexican side additional challenges were present. Testing was severely limited to the point where Mexico was last in testing per capita out of all OECD nations early in the pandemic [41]. This was evident in our data, where mortality rates in Mexico were similar to those in the U.S.; but, case rates in Mexico were on an order of magnitude lower than those in the U.S. This points to a severe lack of testing on the Mexican side, and only limited to symptomatic and sicker individuals. Health infrastructure is severely lacking along the border [42]. Lacking access on the Mexican side of the border is possibly leading many individuals to cross the border to access hospital centers in the U.S. also likely inflating that sides case mortality rates [43]. However, border municipalities have a higher development index then the rest of Mexico. This is partly due to the high number of high-density *maquiladoras (*factories) that manufacture products, including health products for the U.S. market. These *maquiladaroas* were pressured by the U.S. to continue operating. In turn, these *maquiladoras* were early centers for COVID-19 clusters as workers cited not having adequate personal protective equipment and an inability to physically distance within their work environments [44]. Workers’ rights groups have striked as result of these conditions and now that vaccines are available, specific efforts are being made to vaccinate workers in *maquiladoras* [45].

The COVID-19 pandemic had important economic implications in Central America that lead to waves of asylum seekers migrating to the U.S.-Mexico border [15]. Prior to the pandemic, the US instituted a Remain at Home Policy where asylum seekers remained in a third country (in this case Mexico) while their application was being reviewed. This led to refugees remaining along the U.S.-Mexico border in dense migrant camps [15]. Migration across the border has been a point of contention in the political realm of the U.S. with concerns that cases were being imported. While individuals crossing the border were likely not responsible for COVID-19 surges throughout the U.S. [46], the conditions asylum seekers experience are cause for concern and led to difficulties in protecting these individuals from outbreaks in congregate settings [15, 47]. There have been efforts to vaccinate individuals migrating to the U.S. [48], but policies still restrict asylum seekers from entering despite recent opening of the U.S.-Mexico border to vaccinated individuals [49].

Wide sweeping vaccination which cuts across all populations in a society is an essential component to handling any infectious disease. Vaccination for COVID-19 in the first year of availability was variable geographically throughout the U.S. In our data we observed an inverse correlation between vaccination rates and HDI at the border which went against national trends. Possible explanations for these observations lie in states’ and counties’ efforts to target vulnerable socioeconomic groups for increased vaccination resources. Arizona created a subset 1 S which listed groups such as minorities, tribal communities, and uninsured individuals as priorities during all phases of vaccination [50]. California enacted a plan using a Healthy Places Index to divide zip codes throughout the state in four quartiles. Allocation was then conducted 70% by age and 30% by sector with a double allotment for the lowest quartile [50]. New Mexico included a caveat which, based on the CDC’s Social Vulnerability Index (SVI), reallocated up to 25% of supply to higher SVI areas or regions with > 10,000 cases per 100,000 [50]. Texas’ plan included identification of vulnerable groups such as minorities, those experiencing homelessness, and those with disabilities who could contribute to the vaccine allocation decision making process [50]. However, after initial vaccine rollout, local Texas jurisdictions argued that vaccination clinics were predominantly located in wealthier and whiter areas [51–53]. Dallas attempted to modify plans and target Black and Latino neighborhoods; however Texas health officials stated this practice was unacceptable and threatened to cut off all supply [52]. Houston and Austin also enacted policies which identified vulnerable areas based on socioeconomic status, zip code, and COVID-19 burden [54, 55]. With more supply than demand, Texas health officials voted to no longer allocate vaccinations starting on May 10, 2021, and instead created a system in which providers could order shipments [56]. As of writing, many of the racial gaps in vaccination have narrowed or closed [57]. Of note, it is to the authors’ knowledge that many Mexican nationals or residents crossed into the U.S. to get vaccinations early during the vaccination rollout [58]. Potential reasons included inability to access vaccines in Mexico or to access vaccines that were not available in Mexico (e.g., Pfizer or Moderna) at the time. The magnitude of this phenomena is unknown and may overestimate vaccination coverage, especially in the border regions.

On the Mexican side of the border, vaccination began with a rapid start but since slowed. The country was first in Latin America to begin vaccination and doses administered peaked in July. However, poor supply chain and mismanagement of planning seem to have plagued further coverage [59]. Based on our analysis we observed that states along the U.S. border had higher ratios of vaccine doses. This information correlates with the broad push to vaccinate border residents and reopen border traffic and commerce. The U.S. government offered a multitude of doses to support the northern region of Mexico [60]. Additionally, starting in November, vaccines were offered to migrants as a part of the Remain in Mexico program [61].

Limitations of our study lie in the data sourcing and how representative it is of COVID-19 morbidity and mortality. We hoped to avoid testing limitations by focusing specifically on mortality. However, due to healthcare access and public health policies individuals may not be counted or missed [62]. Additionally, the methods through which counties, municipalities, states, and countries count deaths due to COVID-19 vary and can be incredibly complex [63]. When investigating reasons for disparities in COVID-19 outcomes, our metrics for socioeconomic conditions were not perfect comparisons due to differing education systems in the U.S. and Mexico and many other variables likely play a role into COVID-19 related mortality. For example, forced migration is an important factor in healthcare disparities and outcomes that was not assessed. Lastly, Mexican vaccination data more granular than national statistics are severely limited which impacts our ability to investigate its contribution to COVID-19’s spread in the region.

## Conclusions

Overall, border residents and migrants are frequently placed in a precarious situation of handling diseases in a disadvantaged setting for a variety of reasons. Interventions are needed to avoid further mortality due to COVID-19 or future pandemics. Healthcare infrastructure in the forms of treatment, testing, and vaccination is needed, especially on the Mexican side of the border. Further, siloing binational communities is not the answer to avoid excess mortality in the border region. The border has an important interplay that is beneficial to communities on both sides of the border, where curtailing these relationships may ultimately do more harm than good. We believe that coordination from public health officials and policy makers is key to avoid repeating the consequences that these areas have suffered early in the epidemic. These coordinated efforts should be spearheaded by a group of binational leaders who understand the border region, such as the U.S.-Mexico Border Health Commission, instead of distant leaders in state capitals. Only a coordinated effort between both sides of the border will have an impact. Disease activity on a border cities counterpart poses more risk than the overall disease burden of a state, and if concerted efforts are not taken in unison, the other city will always be a reservoir for continued disease transmission and continued case clusters, and ultimately deaths.

## Data Availability

All data and code to run the analysis are available at: https://github.com/davigood1/Border-Covid-gh.

## Abbreviations

SMR: Standardized mortality ratio
HDI: Human Development Index
IRB: Institutional Review Board
ACS: American Community Survey
INEGI: Instituto Nacional de Estadísticia y Geografía
UNDP: United Nations Development Program
